# Targeting MicroRNA-485-3p Blocks Alzheimer’s Disease Progression

**DOI:** 10.3390/ijms222313136

**Published:** 2021-12-04

**Authors:** Han Seok Koh, SangJoon Lee, Hyo Jin Lee, Jae-Woong Min, Takeshi Iwatsubo, Charlotte E. Teunissen, Hyun-Jeong Cho, Jin-Hyeob Ryu

**Affiliations:** 1 BIORCHESTRA Co., Ltd., 17, Techno 4-ro, Yuseong-gu, Daejeon 34013, Korea; kohhan@biorchestra.com (H.S.K.); hohosaraly@biorchestra.com (H.J.L.); mjw@biorchestra.com (J.-W.M.); 2 Department of Infection Biology, Faculty of Medicine, University of Tsukuba, Ibaraki 305-8577, Japan; frank.sj.lee@gmail.com; 3 Department of Neuropathology, Graduate School of Medicine, The University of Tokyo, Tokyo 113-0033, Japan; iwatsubo@m.u-tokyo.ac.jp; 4 Neurochemistry Laboratory and Biobank, Department of Clinical Chemistry, Amsterdam Neuroscience, Amsterdam University Medical Centers, 1081 HV Amsterdam, The Netherlands; c.teunissen@amsterdamumc.nl; 5 Department of Biomedical Laboratory Science, College of Medical Science, Konyang University, Daejeon 35365, Korea; 6 BIORCHESTRA US Inc., 245 Main St., Cambridge, MA 02142, USA

**Keywords:** Alzheimer’s disease, β-amyloid, tau, IL-1β, TNF-α, neuroinflammation, cognitive function, microRNA, miR-485-3p, antisense oligonucleotide

## Abstract

Alzheimer’s disease (AD) is a form of dementia characterized by progressive memory decline and cognitive dysfunction. With only one FDA-approved therapy, effective treatment strategies for AD are urgently needed. In this study, we found that microRNA-485-3p (miR-485-3p) was overexpressed in the brain tissues, cerebrospinal fluid, and plasma of patients with AD, and its antisense oligonucleotide (ASO) reduced Aβ plaque accumulation, tau pathology development, neuroinflammation, and cognitive decline in a transgenic mouse model of AD. Mechanistically, miR-485-3p ASO enhanced Aβ clearance via CD36-mediated phagocytosis of Aβ in vitro and in vivo. Furthermore, miR-485-3p ASO administration reduced apoptosis, thereby effectively decreasing truncated tau levels. Moreover, miR-485-3p ASO treatment reduced secretion of proinflammatory cytokines, including IL-1β and TNF-α, and eventually relieved cognitive impairment. Collectively, our findings suggest that miR-485-3p is a useful biomarker of the inflammatory pathophysiology of AD and that miR-485-3p ASO represents a potential therapeutic candidate for managing AD pathology and cognitive decline.

## 1. Introduction

The amyloid-beta (Aβ) hypothesis postulates that the accumulation of neurotoxic cleaved Aβ proteins is the primary cause of synaptic dysfunction and the subsequent neurodegeneration that underlies the characteristic progression of Alzheimer’s disease (AD) [1,2]. Genetic and neuropathological evidence suggests that targeting Aβ plaque formation can be beneficial for patients with AD [3,4]. Currently, there is only one FDA-approved therapy for AD despite its increasing global incidence; thus, effective treatment strategies for AD are urgently needed. While several drugs that decrease Aβ production or increase Aβ clearance in the brain have been identified, treatment with these drugs is poorly correlated with improvements in AD severity and cognitive dysfunction [5,6,7,8,9,10,11].

The neuropathological hallmarks of AD are widespread deposition of Aβ plaques in the neocortex and a hierarchically organized pattern of neurofibrillary tangles composed of hyperphosphorylated and cleaved tau aggregates in limbic and cortical association areas [12,13,14]. Additionally, emerging evidence has shown that microglia, resident immune cells of the brain, play a potentially detrimental role by eliciting the expression of proinflammatory cytokines influencing neurodegeneration in AD [15,16,17,18]. Among the proinflammatory cytokines, interleukin-1β (IL-1β) is a key mediator of the inflammatory response that stimulates AD pathology [15,16,17]. IL-1β is controlled through inflammasome-mediated proteolytic maturation of pro-IL-1β. Inflammasomes are multiprotein complexes consisting of caspase-1, ASC, and cytoplasmic pathogen recognition receptors (PRRs) such as nucleotide-binding oligomerization domain (NOD)-like receptor family proteins (NLRPs) and absent in melanoma 2 (AIM2)-like receptors (ALRs) [19,20,21,22,23,24].

MicroRNAs (miRNAs), as noncoding single-stranded RNAs, have been shown to function as regulators of development, growth, differentiation, and neurodegenerative processes [25]. A single miRNA molecule can regulate various genes, making miRNAs a potential therapeutic target for multifactorial conditions, such as brain disease. Indeed, miRNAs orchestrate a variety of signaling pathways involved in AD pathogenesis, including those involved in Aβ production and clearance, neuroinflammation, and neurogenesis [26,27,28,29]. However, the molecular mechanisms of miRNAs in AD and their potential for use in AD therapy are poorly understood.

Here, we found that the microRNA-485-3p (miR-485-3p) was overexpressed in the brain tissues and cerebrospinal fluid (CSF) of patients with AD, and its antisense oligonucleotide (ASO) reduced Aβ plaque accumulation, tau pathology development, neuroinflammation, and cognitive decline in a transgenic mouse model of AD. Mechanistically, miR-485-3p ASO enhanced Aβ clearance via the CD36-mediated phagocytosis of Aβ in vitro and in vivo. We also found that miR-485-3p ASO reduced apoptosis, which effectively decreased truncated tau levels. Further, miR-485-3p ASO reduced the secretion of proinflammatory cytokines, including IL-1β and TNF-α, and eventually relieved cognitive impairment. Collectively, our findings suggest that miR-485-3p is a useful biomarker of the inflammatory pathophysiology of AD. Furthermore, miR-485-3p ASO may represent a therapeutic candidate for AD pathology and cognitive decline, thereby establishing a new paradigm in the AD field.

## 2. Results

### 2.1. miR-485-3p Is Overexpressed in Alzheimer’s Disease Patients

**Figure 1:**
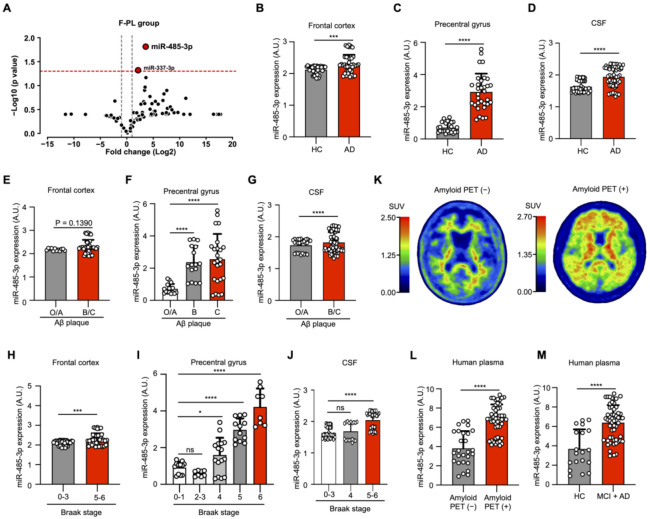
miR-485-3p is overexpressed in Alzheimer’s disease patients. (**A**) Results of differentially expressed miRNA analysis using founder-plasma samples from patients with AD (all patients were amyloid-PET positive, *n* = 3) and healthy controls (all subjects were amyloid-PET negative, *n* = 3). The red horizontal dotted line indicates *p* = 0.05. The two grey vertical dotted lines represent −1 and +1. Eighty-four miRNAs were normalized using 8 reference genes. (**B**–**D**) Expression of miR-485-3p in the human frontal cortex by real-time PCR (healthy control [HC] *n* = 8; patients with AD *n* = 7) (**B**), precentral gyrus (healthy control [HC] *n* = 6; patients with AD *n* = 8) (**C**), and cerebrospinal fluid (CSF) (healthy control [HC] *n* = 6; patients with AD *n* = 7) (**D**). Data obtained across three independent experiments are expressed as mean ± SD. (**E**–**G**) Expression patterns of miR-485-3p in the frontal cortex by real-time PCR (O/A *n* = 2; B/C *n* = 7) (**E**), precentral gyrus (O/A *n* = 4; B/C *n* = 10) (**F**), and CSF (O/A *n* = 3; B/C *n* = 10), (**G**) depending on the level of Aβ plaque accumulation. Level O/A means no or low densities of amyloid deposits in the isocortex, particularly in the basal portions of the frontal, temporal, and occipital lobes. Level B shows an increase in amyloid deposit levels in almost all isocortical association areas; only the primary sensory areas and primary motor field remained almost devoid of deposits. Level C is mainly characterized by virtually all isocortical areas being affected; deposits in the hippocampus show the same pattern as those in Level B. Data obtained across three independent experiments are expressed as mean ± SD. (**H**–**J**) Expression patterns of miR-485-3p in the frontal cortex by real-time PCR (stages 0 to 3: *n* = 8; stages 5 to 6 *n* = 7) (**H**), precentral gyrus (stages 0 to 1: *n* = 3; stages 2 to 3: *n* = 2; stages 4: *n* = 4; stage 5: *n* = 3; stage 6: *n* = 2) (**I**), and CSF (stages 0 to 3: *n* = 6; stages 4: *n* = 2; stages 5 to 6: *n* = 5) (**J**), depending on Braak stage. Data obtained across three independent experiments are expressed as mean ± SD. (**K**,**M**) Amyloid-PET images and standard uptake values (SUVs) of two clinical samples. Each SUV is displayed as a rainbow bar on the left. The images were obtained using a GE Discovery PET/CT 690 as a scanner and florbetaben as a tracer (**K**). Expression of miR-485-3p in plasma exosomes according to amyloid PET images (amyloid-PET-negative patients *n* = 14; amyloid-PET-positive patients *n* = 25) (**L**). Data obtained across three independent real-time PCR experiments are expressed as mean ± SD. Expression of miR-485-3p in plasma exosomes according to cognitive diagnostic results (healthy control *n* = 10; MCI patients *n* = 17; patients with AD patients *n* = 12) (**M**). Data obtained across three independent real-time PCR experiments are expressed as mean ± SD. For the frontal cortex (**B**,**E**,**H**), six repeat tests were performed per sample. In the case of the precentral gyrus (**C**,**F**,**I**), four repeat tests were performed per sample. In the case of CSF (**D**,**G**,**J**), nine repeat tests were performed per sample. For plasma (**L**,**M**), an average of 1.92 repeat tests per sample was performed. * *p* < 0.05; *** *p* < 0.001, **** *p* < 0.0001 (two-tailed *t*-test).

To identify the miRNAs involved in the pathogenesis of AD, we performed miRNA screening of plasma samples obtained from patients with AD (*n* = 3) and a healthy control group (*n* = 3). This screening showed that miR-337-3p and miR-485-3p were upregulated in patients with AD, with miR-485-3p having the most upregulated expression (Figure 1A). This finding was consistent with our individual experimental data on miR-485-3p expression in the frontal cortex, precentral gyrus, and cerebrospinal fluid (CSF) of patients with AD (Figure 1B–D). Additionally, the expression patterns of miR-485-3p in the human precentral gyrus and CSF were dependent on the level of Aβ plaque accumulation, although the expression was slightly increased in human frontal cortex (Figure 1E–G) and the AD Braak stage (Figure 1H–J). Aβ positron emission tomography (Aβ PET) scanning, the gold-standard method for confirmatory diagnosis of AD, showed upregulation in patients with mild cognitive impairment (MCI) and AD, which showed a similar appearance to miR-485-3p expression in the plasma exosomes (Figure 1K–M and Figure S1). We found that miR-485-3p expression was upregulated in MCI patients with positive Aβ PET compared to that in MCI patients with negative Aβ PET (Figure S2). Collectively, these data suggest that miR-485-3p is overexpressed in AD patients.

### 2.2. miRNA-485-3p Induces Accumulated Ab Plaque, Phosphorylated Tau, Cleaved Tau, and Reduced Synaptophysin and PSD-95 Conditions in Primary Mouse Neurons

**Figure 2:**
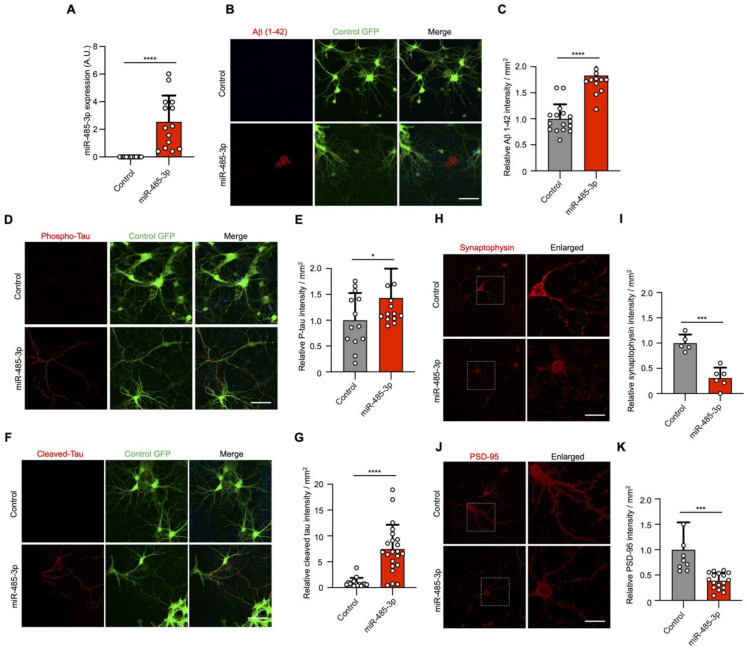
miRNA-485-3p induces accumulated Ab plaque, phosphorylated tau, cleaved tau, and reduced synaptophysin and PSD-95 conditions in primary mouse neurons. (**A**) After lentivirus-derived miR-485-3p transduction, miR-485-3p expression in primary mouse neurons was analyzed by real-time PCR (*n* = 14; data obtained across three independent experiments are expressed as mean ± S.E.M.). (**B**,**C**) Aβ 1–42 plaque immunofluorescence images of primary mouse neurons after lentivirus-derived miR-485-3p transduction (**B**). Scale bars, 20 μm. Data are representative of three independent experiments. Quantification of the Aβ 1–42 intensity (**C**) (two-tailed *t*-test; lenti-control *n* = 16, lenti-miR-485-3p *n* = 17; data are expressed as mean ± S.E.M. representing 1–5 randomly sampled regions, pooled from three independent experiments). (**D**,**E**) Phospho-tau immunofluorescence images of primary mouse neurons after lentivirus-derived miR-485-3p transduction (**D**). Scale bars, 20 μm. Data are representative of three independent experiments. Quantification of the phospho-tau intensity (**E**) (lenti-control *n* = 13, lenti-miR-485-3p *n* = 15; data are expressed as mean ± S.E.M. representing 1–5 randomly sampled regions, pooled from three independent experiments). (**F**,**G**) Cleaved tau immunofluorescence images of primary mouse neurons after lentivirus-derived miR-485-3p transduction (**F**). Scale bars, 20 μm. Data are representative of three independent experiments. Quantification of the cleaved tau intensity (**G**) (two-tailed *t*-test; lenti-control *n* = 15, lenti-miR-485-3p *n* = 22; data are expressed as mean ± S.E.M. representing 1–5 randomly sampled regions, pooled from three independent experiments). (**H**–**K**) Synaptophysin (**H**) or PSD-95 (**J**) immunofluorescence images of primary mouse neurons after lentivirus-derived miR-485-3p transduction. Scale bars, 20 μm. Data are representative of three independent experiments. Quantification of the synaptophysin (**I**) or PSD-95 (**K**) intensity (two-tailed *t*-test; synaptophysin, lenti-control *n* = 5, lenti-miR-485-3p *n* = 6; PSD-95, lenti-control *n* = 9, lenti-miR-485-3p *n* = 17; data are expressed as mean ± S.E.M. representing 1–5 randomly sampled regions, pooled from three independent experiments). * *p* < 0.05; *** *p* < 0.001; **** *p* < 0.0001 (two-tailed *t*-test).

To investigate whether miR-485-3p is a neuropathological miRNA in AD, we examined AD pathology in miR-485-3p-transduced primary murine neurons (Figure 2A). Aβ plaque accumulation was observed in the extracellular space after miR-485-3p transduction (Figure 2B,C). Tau hyperphosphorylation and cleaved tau accumulation were induced in primary murine neurons after miR-485-3p transduction (Figure 2D–G). We also found a significant decrease in the levels of presynaptic synaptophysin and postsynaptic density protein PSD-95 after miR-485-3p transduction (Figure 2H–K). These data indicate that miR-485-3p is causally involved in AD pathology. We next sought to see whether miR-485-3p expression could induce inflammasome activation and IL-1β release. Indeed, the number of ASC specks and release of IL-1β in primary murine microglia increased after miR-485-3p transduction (Figure S3), suggesting that miR-485-3p induces the inflammatory response in AD.

### 2.3. miR-485-3p ASO Reduces Aβ Pathology and Neuroinflammation and Rescues Cognitive Impairment

**Figure 3:**
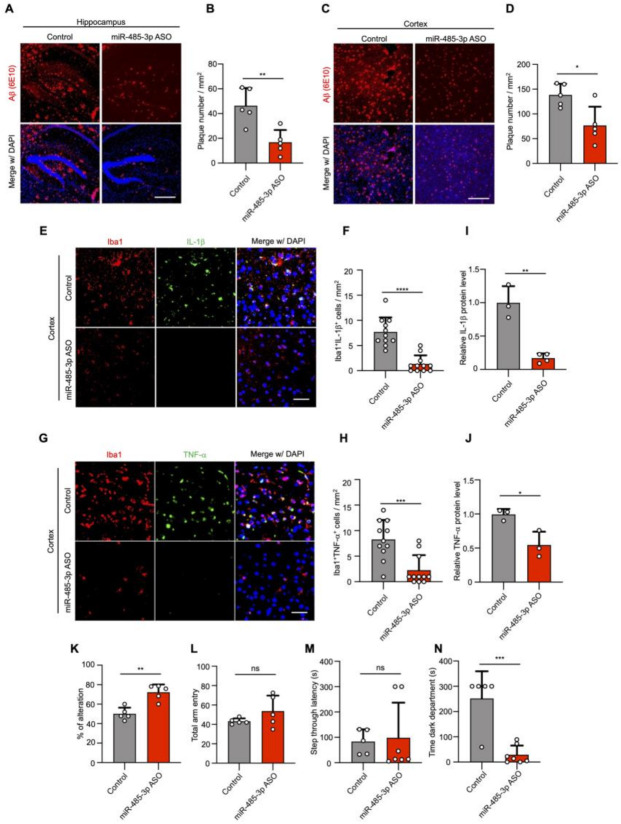
miR-485-3p antisense oligonucleotide (ASO) reduces Aβ pathology and neuroinflammation and rescues cognitive impairment. (**A**–**D**) Representative images of immunohistochemical staining with Aβ (6E10) in the hippocampus (**A**) and cortex region (**C**) after either control oligonucleotide or miR-485-3p ASO injection. Scale bars, 300 μm. Quantification of the Aβ plaque in the hippocampus (**B**) and cortex region (**D**) (two-tailed *t*-test; *n* = 5; data are expressed as mean ± SD representing 1–3 randomly sampled regions, pooled from two independent experiments). (**E**) Representative images of immunohistochemical staining for Iba1 and IL-1β in the cortex region of control- or miR-485-3p ASO-injected 8-month-old 5XFAD mice. Scale bars, 20 μm. (**F**) Quantification of the Iba1^+^IL-1β cells from (**E**) (two-tailed *t*-test; *n* = 11; data are expressed as mean ± SD representing 1-4 randomly sampled regions, pooled from three independent experiments). (**G**) Representative images of immunohistochemical staining for Iba1 and TNF-α in the cortex region of control- or miR-485-3p ASO-injected 8-month-old 5XFAD mice. Scale bars, 20 μm. (**H**) Quantification of the Iba1+ TNF-α cells from (**G**) (two-tailed *t*-test; *n* = 11; data are expressed as mean ± SD representing 1-4 randomly sampled regions, pooled from three independent experiments). (**I**,**J**) Protein expression of IL-1β (**I**) and TNF-α (**J**) in the cortex of control- or miR-485-3p ASO-injected 5XFAD mice (data obtained across three independent experiments are expressed as mean ± SD). (**K**–**N**) Behavior tests in control- (*n* = 5–7) or miR-485-3p ASO- (*n* = 5–7) injected 8-month-old 5XFAD mice. Y-maze (**K**,**L**) analysis and passive avoidance testing (**M**,**N**) of control- or miR-485-3p ASO-injected 8-month-old 5XFAD mice. Average alternation (%) (**K**) for control- or and miR-485-3p ASO-injected 5XFAD mice and total entry number (**L**) into each arm on the Y-maze. Average step-through latency (**M**) and time in the dark compartment in seconds (**N**) for control- and miR-485-3p ASO-injected 5XFAD mice in passive avoidance tests. All data are presented as mean ± SD. ns, not significant; * *p* < 0.05; ** *p* < 0.01; *** *p* < 0.001; **** *p* < 0.0001 (two-tailed *t*-test).

To understand the potential of miR-485-3p as a therapeutic target in AD, we synthesized miR-485-3p antisense oligonucleotides (ASO) and examined whether the pharmacological inhibition of miR-485-3p suppresses AD pathology in vivo. We first confirmed that there was no difference in the level of miR-485-3p associated with the number of months in WT mice (Figure S4A). However, in 5XFAD mice, the 11-month-old mice showed increased levels of 485-3p compared to that in the 6-month-old mice (Figure S4B), suggesting that miR-485-3p expression increases with age in the 5XFAD mice. The miR-485-3p ASO formulated with jetPEI reagent in vivo was injected into the right lateral ventricle of 5XFAD transgenic mice by stereotaxic injection, and the injections were repeated on day 7. As previously reported [30,31,32], 5XFAD transgenic mice stained with 6E10 showed Aβ plaque deposition and Aβ aggregation in insoluble clusters or plaque formation in the hippocampus and cortex with AD progression (Figure 3A–D). 

In contrast, the number of Aβ plaques was markedly decreased in 5XFAD mice administered intracerebroventricular injections of miR-485-3p ASO (Figure 3A–D), suggesting that miR-485-3p ASO ameliorated the Aβ burden. Insoluble Aβ 1–42 production also decreased in the cortex of 5XFAD mice after miR-485-3p ASO injection, compared with that in the cortex of control-injected 5XFAD mice (Figure S5A,B). While total APP expression was comparable, the expressions of β-CTFs and sAPPβ in the cortex of miR-485-3p ASO-injected 5XFAD mice were reduced, compared with those in the cortex of control-injected 5XFAD mice (Figure S5C–G), indicating that the miR-485-3p ASO triggered strong reductions in both Aβ production and plaque formation in vivo. 

Immunohistochemistry with Iba1 (microglia-specific calcium-binding protein) and antibodies against IL-1β or TNF-α showed that IL-1β and TNF-α levels in microglia were markedly attenuated in miR-485-3p ASO-injected 5XFAD mice (Figure 3E–H). Similar results were obtained for the protein expression of IL-1β and TNF-α in the cortex of miR-485-3p ASO-injected 5XFAD mice (Figure 3I,J), suggesting that the miR-485-3p ASO reduced proinflammatory responses via microglial inactivation. 

We next examined cognitive function in control- and miR-485-3p ASO-treated 8-month-old 5XFAD mice using a Y-maze and passive avoidance tasks (PATs), which are widely accepted as behavior paradigms for evaluating spatial working memory [33]. Two days after the last miR-485-3p ASO injection, the spontaneous alternation percentage was increased in miR-485-3p ASO-injected 5XFAD mice (Figure 3K). The total number of arm entries did not differ between control- and miR-485-3p ASO-treated 5XFAD mice (Figure 3L), indicating that the levels of the general motor and exploratory activities in the Y-maze assessment were not changed. 

In addition, we examined associative memory in the PAT based on the association formed between an electrical foot shock and a spontaneously preferred environmental context (darkness vs. light). Step-through latency was similar between control- and miR-485-3p ASO-treated 5XFAD mice (Figure 3M). However, the miR-485-3p ASO mice showed a reduction in the latency of time spent in the dark compartment, 24 h after receiving an electrical shock (Figure 3N). These results suggest that the miR-485-3p ASO reduced Aβ accumulation and neuroinflammation and rescued cognitive impairment. 

**Figure 4:**
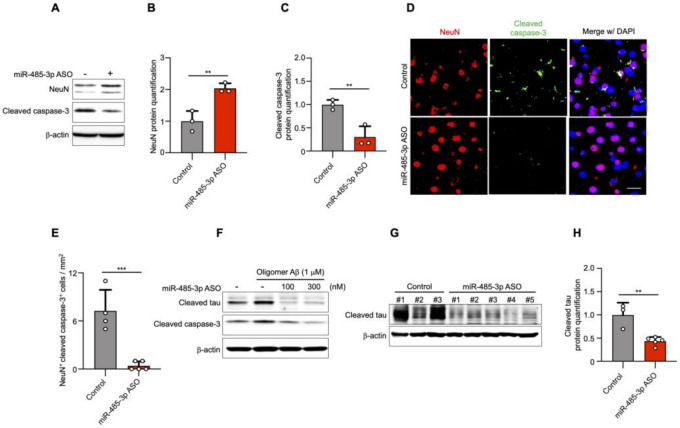
miR-485-3p antisense oligonucleotide (ASO) reduces apoptosis and truncated tau level. (**A**) Immunoblotting for NeuN and cleaved caspase-3 expression in fractions from the cortex region of control- or miR-485-3p ASO-injected 10-month-old 5XFAD mice. Data are representative of three independent experiments. (**B**,**C**) Relative protein quantification of NeuN (**B**) and cleaved caspase-3 (**C**) obtained from (**A**). Data obtained across four independent experiments are expressed as mean ± SD. (**D**) Immunohistochemical staining for NeuN and cleaved caspase-3 in coronal brain sections from control- or miR-485-3p ASO-injected 10-month-old 5XFAD mice. Scale bars, 20 μm. Data are representative of three independent experiments. (**E**) Quantification of the NeuN^+^cleaved caspase-3^+^ cells from control- (*n* = 4) or miR-485-3p ASO- (*n* = 5) injected 10-month-old 5XFAD mice. (**F**) Primary cortical neurons were treated with 1 μM oligomeric Aβ (1–42) for 6 h; lysates were immunoblotted with cleaved tau or cleaved caspase-3 antibodies. Data are representative of three independent experiments. (**G**) Immunoblotting for cleaved tau protein expression in control- (*n* = 3) or miR-485-3p ASO- (*n* = 5) injected 10-month-old 5XFAD mice. (**H**) Relative protein quantification of cleaved tau obtained from (**G**). Data obtained over three independent experiments are expressed as mean ± SD. ** *p* < 0.01; *** *p* < 0.001 (two-tailed *t*-test).

The 5XFAD transgenic mice show neuronal loss in cortical layer V at 4 and 9 months [30]. We next investigated the effect of administering an antisense inhibitor on neuronal cell death by assessing NeuN (a neuronal cell marker) and cleaved caspase-3. The expression of NeuN was increased, but that of cleaved caspase-3 was decreased in the cortical region of 10-month-old miR-485-3p ASO-treated 5XFAD mice (Figure 4A–C).

Immunohistochemistry showed that the expression of cleaved caspase-3 in neuronal cells was markedly decreased in miR-485-3p ASO-treated 5XFAD mice compared with that in control-treated 5XFAD mice (Figure 4D,E). To elucidate whether the reduction in cleaved caspase-3 levels was associated with cleaved tau, primary murine cortical neurons were simultaneously transfected with miR-485-3p ASO and treated with oligomeric Aβ. The activation of both cleaved caspase-3 and cleaved tau decreased in a miR-485-3p ASO dose-dependent manner (Figure 4F). Cleaved tau expression was also decreased in the brains of miR-485-3p ASO-injected 10-month-old 5XFAD mice (Figure 4G,H). These results suggested that the miR-485-3p ASO reduced apoptosis, effectively reducing tau pathology in AD.

### 2.4. miR-485-3p ASO Enhances the Phagocytosis of Aβ by Regulating CD36 Expression In Vitro and In Vivo

**Figure 5:**
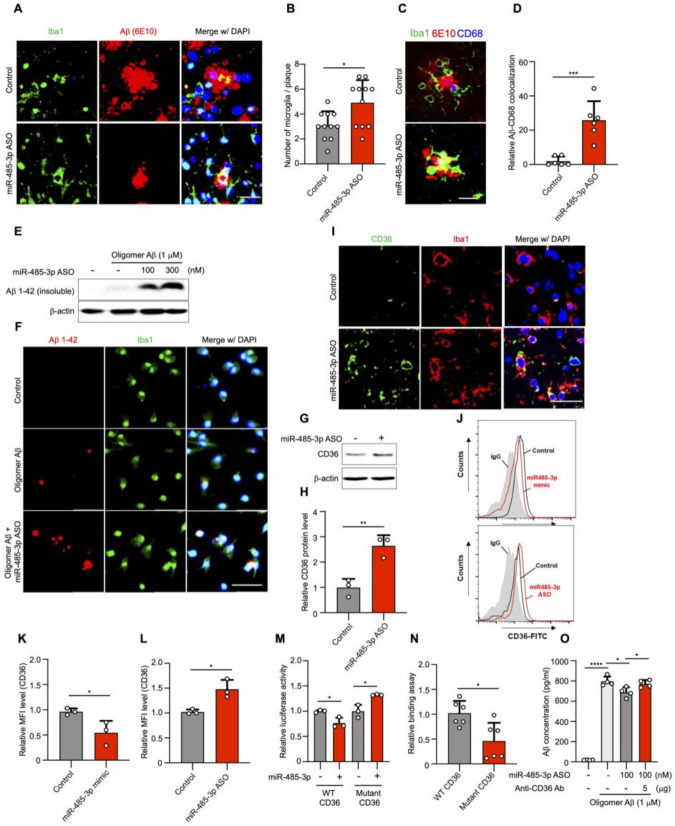
miR-485-3p antisense oligonucleotide enhances phagocytosis of Aβ by regulation of CD36 in vitro and in vivo. (**A**) Representative images of immunohistochemical staining for Iba1 and Aβ (6E10) in the cortex region of control- and miR-485-3p ASO-injected 8-month-old 5XFAD mice. Data are representative of five independent experiments. (**B**) Quantification of Iba1^+^Aβ^+^ cells from (**A**) (control *n* = 13, miR-485-3p ASO *n* = 11 from five biologically independent samples). (**C**) Representative images of immunohistochemical staining for Iba1, CD68 (phagosome), and Aβ (6E10) in the cortex region of control- and miR-485-3p ASO-injected 8-month-old 5XFAD mice. Data are representative of three independent experiments. (**D**) Quantification of the Iba1^+^CD68^+^Aβ^+^ cells from (**C**) (control *n* = 6, miR-485-3p ASO *n* = 6 from three biologically independent samples). (**E**) Control- or miR-485-3p ASO-transfected BV2 microglia treated with oligomer Aβ (1–42). After 4 h, the Ab insoluble fraction was analyzed by immunoblotting for analysis of uptake capacity. Data are representative of three independent experiments. (**F**) Primary mouse microglia were transfected with control or miR-485-3p ASO and treated with oligomer Aβ (1–42). After 4 h, the cells were examined by immunocytochemistry using Iba1 and 6E10 antibodies. Data are representative of three independent experiments. (**G**) CD36 protein expression in the cortex region of control- or miR-485-3p ASO-injected 8-month-old 5XFAD mice. Data are representative of three independent experiments. (**H**) Quantification of CD36 protein expression from (**G**). Data obtained across three independent experiments are expressed as mean ± SD. (**I**) Representative images of immunohistochemical staining with Iba1 and CD36 in coronal brain sections of control- or miR-485-3p ASO-injected 8-month-old 5XFAD mice. Data are representative of three independent experiments. (**J**–**L**) Cell surface expression of CD36 was analyzed by flow cytometry using Alexa488-conjugated anti-CD36 antibody in control-, miR485-3p mimic-, or miR-485-3p ASO-transfected primary glial cells (**J**). Data are representative of three independent experiments. Relative quantification of CD36 expression in control and miR-485-3p (**K**) or control and miR-485-3p ASO (**L**) obtained from (**J**). Data obtained across three independent experiments are expressed as mean ± SD. (**M**) Relative luciferase activity in HEK293T cells co-transfected with CD36 3′-UTR WT or mutant reporter constructs and miR-control or miR-485-3p for 48 h. Data obtained across three independent experiments are expressed as mean ± SD. (**N**) Relative binding of miR-485-3p to the 3′ UTR of CD36 harboring mutant seed sequences, compared with those binding to the 3′ UTR of WT CD36. (**O**) Control- or miR-485-3p ASO-transfected microglial cells were treated with 1 μM oligomer Aβ (1–42) with IgG or a CD36-blocking antibody. After 4 h, the supernatant was analyzed using ELISA for phagocytosis. Data obtained across three independent experiments are expressed as mean ± SD. * *p* < 0.05; ** *p* < 0.01; *** *p* < 0.001; **** *p* < 0.0001 (two-tailed *t*-test).

We next sought to characterize the mechanism through which the miR-485-3p ASO reduced Aβ plaque formation. Microglia have been characterized as mediating the clearance and phagocytosis of aggregated Aβ in AD brains, thereby contributing to the alleviation of AD symptoms [34,35,36], but whether this also occurs upon injecting miR-485-3p ASO is unknown. Immunohistochemical staining for Iba1 and Aβ (6E10) showed that the expression of Aβ in microglia was elevated in miR-485-3p ASO-treated 8-month-old 5XFAD mice (Figure 5A,B). To assess Aβ engulfment and clearance by glial cells, we quantified CD68-positive microglial phagosomes and internalized Aβ. The clustering of Iba1-positive microglia surrounding amyloid plaques indicated a diffuse CD68 distribution in miR-485-3p ASO-treated 8-month-old 5XFAD mice, compared with that in control mice (Figure 5C,D). To further assess the phagocytic effect of miR-485-3p ASO in microglia, we treated miR-485-3p ASO-transfected microglia with oligomer Aβ 1−42. Aβ uptake by microglia increased in a miR-485-3p ASO-dose dependent manner (Figure 5E,F), indicating that the miR-485-3p ASO enhanced Aβ phagocytosis in microglia.

CD36 contributes to Aβ phagocytosis in microglia [37,38,39]. To elucidate whether CD36 contributed to Aβ phagocytosis upon treatment with miR-485-3p ASO, we examined the expression of CD36 in 5XFAD mice. The protein expression of CD36 in the brain (Figure 5G,H) or Iba1-positive microglia (Figure 5I) was higher in miR-485-3p ASO-injected 8-month-old 5XFAD mice than in control mice. CD36 expression was decreased in miR-485-3p-transfected primary glial cells, compared with that in control cells (Figure 5J,K); miR-485-3p ASO transfection also elevated CD36 expression, compared with the expression in the control (Figure 5J,L). 

We next examined miR-485-3p targeting of the 3′-UTR of CD36 using reporter plasmids containing either wild-type or mutant forms of the potential miR-485-3p target site. The plasmid containing the wild type (WT) CD36 3‘UTR reduced luciferase activity, whereas the plasmid containing the mutant form of the target site did not (Figure 5M). miR-485-3p directly regulated luciferase activity by binding to the CD36 3′-UTR, but its binding efficiency was reduced for the 3′ UTR of mutant CD36, compared with that for the 3′ UTR of the wild type (Figure 5N). In addition, Aβ levels in the extracellular space were decreased in miR-485-3p ASO-transfected cells, compared with those in control-transfected cells; this effect was abrogated in cells treated with a CD36 blocking antibody (Figure 5O). These results suggest that the miR-485-3p ASO regulated CD36 expression, which in turn affected Aβ phagocytosis.

## 3. Discussion

We found that miR-485-3p played a crucial role in the formation of Aβ plaques, tau pathology development, and upregulation of inflammatory response, and may affect cognitive decline in AD. Furthermore, miR-485-3p was overexpressed in patients with AD, and it inhibited microglial Aβ phagocytosis by directly interacting with CD36, resulting in impaired Aβ clearance during AD progression; these results suggest that miR-485-3p acts as a biomarker of AD that triggers AD pathology.

Yu et al. recently reported that miR-485-3p serves as a biomarker and therapeutic target of AD [40]. They showed that serum miR-485-3p expression was significantly upregulated in AD patients Furthermore, they present that knockdown of miR-485-3p in SH-SY5Y cells improves neuronal proliferation and decreases cell apoptosis after Aβ treatment. In BV2 cells, they show that knockdown of miR-485-3p reduces levels of IL-1β, IL-6, and TNF-alpha after Aβ treatment. However, their study appears to have been conducted at a rather preliminary stage; they examined levels of miR-485-3p in the human serum but not in the frontal cortex, precentral gyrus, or CSF. Additionally, their study mainly examined the effects of miR-485-3p in cell-based in vitro systems, and there was no in vivo physiological relevance to their study. Therefore, we firmly believe that our work reveals the molecular basis underlying the triggering of AD pathology by miR-485-3p in vitro and in vivo. 

We also highlight that treatment with miR-485-3p ASO reduces AD pathology. The failures of clinical trials do not disprove the Aβ hypothesis; instead, they highlight the need to consider the highly complex environment of AD. Experimental and clinical findings indicate that, other than Aβ clearance, reduced tau deposition and neuroinflammation play crucial roles in the pathogenesis of AD [15,16,17,41,42]. Given the urgent requirement for effective treatment strategies for AD, miR-485-3p ASO represents a therapeutic candidate for reducing Aβ plaque accumulation, tau pathology development, neuroinflammation, and cognitive decline.

Overall, our findings indicate that miR-485-3p is a trigger for AD pathology progression and define the molecular basis of miR-485-3p ASO-mediated reduction of Aβ plaque and cleaved tau as well as neuroinflammation in AD. Understanding the complex molecular underpinnings of miR-485-3p in AD is critical to identifying new therapeutic strategies and possible ways to counteract this deadly disease.

## 4. Materials and Methods

### 4.1. Human Tissue

Brain frontal cortex, precentral gyrus, and CSF samples from patients with AD and control subjects were purchased from the Netherlands Brain Bank (NBB, KYUH 2016-11-024-003). Detailed information on the samples is provided in Tables S1–S3.

### 4.2. Sample for miRNA Screening 

Founder-plasma samples (F-PLs) were the plasma samples used to screen for microRNA biomarkers. Reproducibility check-plasma exosomes (RC-PLEs) were the sample group used to assess reproducibility in the plasma exosome state of a microRNA biomarker.

Human-derived plasma samples used in F-PL were prospectively collected with Institutional Review Board (IRB) approval from Konyang University Hospital (KYUH-2016-11-024). Human-derived plasma samples used in RC-PLE were prospectively collected with the approval from the Institutional Review Boards of the Boramae Medical Center (approval number 20180508/10-2018-53/061), the Eulji Medical Center (approval number EMC 2019-06-036-002), and the Gyeongsang National University Hospital (approval number GNUH 2019-07-026-002) in South Korea. Detailed sample information is provided in Table S4.

### 4.3. qPCR Array and Screening of Differentially Expressed miRNAs (DEmiRNAs)

Approximately 3 mL of blood was collected in a blood tube containing sodium citrate (3.2% *w*/*v*) from 4 patients diagnosed with AD by physicians and 4 healthy adults (Table S5). Blood was centrifuged at 3500 rpm for 10 min to separate plasma and stored at −80 °C until miRNA extraction. miRNA was extracted using the miRNeasy Serum/Plasma kit (Qiagen, Germantown, MD 20874, USA) according to the manufacturer’s recommendations. The concentration and purity of the extracted RNA were measured using Bioanalyzer 2100 (Agilent, Santa Clara, CA 95051, USA). Eight samples met the quality criteria and were used in the study. Data quality control (QC) was performed according to the guidelines provided by QIAGEN Co., Ltd (Venlo, The Netherlands). Two samples (PL_N6 and PL_AD2) were excluded from the analysis based on the results of four RNA controls (H9 to H12 in Table S6). Based on positive PCR control (PPC), all samples showed a “Failed” result. Since only two samples were excluded based on reverse transcription control (RTC), sample QC was performed based on RTC. Finally, 3 healthy control (PL_N1, PL_N2, and PL_N3) and 3 AD (PL_AD1, PL_AD3, and PL_AD4) samples were used for screening DEmiRNAs. If the cycle threshold (Ct) value of PCR was not measured, or the Ct value was over 35, the Ct value was substituted with 35. The PCR results of miRNAs were normalized with the average value of 8 RNA controls (H1 to H8 in Table S6). Formula: Normalized expression = 2(Average Ct of standard—Ct of each miRNA) × 10^3^. The *p*-value was obtained using the “edgeR” package of R (version 4.0.0). We finally selected several miRNAs with a *p*-value of 0.05 or less as DEmiRNAs.

### 4.4. Mice

B6SJLF1/J (JAX#100012) and five familial AD mutation (5XFAD) transgenic mice (#MMRRC#034848) were purchased from The Jackson Laboratory (Bar Harbor, ME, USA). The 5XFAD mice overexpressed mutant human amyloid precursor protein (APP) with the Swedish (K670N, M671L), Florida (I716V), and London (V717I) mutations along with mutant human presenilin 1 (PS1), which carried two FAD mutations (M146L and L286V). These transgenes are regulated by the Thy1 promoter in neurons. The genotype of the 5XFAD mice was confirmed by PCR analysis of tail DNA in standard conditions and provided by The Jackson Laboratory. Mice of mixed genotypes were housed at four to five mice per cage in a 12 h light/dark cycle with food and water provided ad libitum. All animal procedures were performed according to the Konyang University guidelines for animal care (Permit number: P-18-18-A-01).

### 4.5. Intraventricular Injection of miR-485-3p ASO

miR-485-3p ASO (AGAGAGGAGAGCCGUGUAUGAC) was synthesized by Integrated DNA Technologies (Coralville, IA, USA). The non-targeting ASO (negative control, #AM17010) was purchased from Thermo Fisher (Waltham, MA, USA). All animals were initially anesthetized with 3–5% isoflurane in oxygen and fixed on a stereotaxic frame (JeongDo, Paju, Korea). For intracerebroventricular (ICV) injection, both the miR-485-3p ASO samples and the non-targeting control oligonucleotides were formulated with the jetPEI reagent (Polyplus, Berkeley, CA, USA) in vivo. The miR-485-3p ASO (1.5 μg) or control oligonucleotides formulated with the jetPEI reagent were injected using a 10 μL Hamilton syringe (26-gauge blunt needle) at 1.5 μL/min. The miR-485-3p ASO were infused in a volume of 5 μL into 8- or 10-month-old 5XFAD mice via ICV injection, and both the ASO and non-targeting control oligonucleotides were administered once weekly for 2 weeks. The intracerebroventricular (ICV) position was identified using the coordinates from the bregma: AP = −0.2 mm, L = ±1.0 mm, ventral (V) = −2.5 mm.

### 4.6. Cell Culture and Transfection

Mouse primary glial cells were cultured from the cerebral cortices of 1- to 3-day-old C57BL/6 mice. The cerebral cortex was dissected and triturated into single-cell suspensions by pipetting. Then, the single-cell suspensions were plated into 6-well plates pre-coated with 0.05 mg/mL poly-D-lysine (PDL) and cultured in DMEM supplemented with 25 mM glucose, 10% fetal bovine serum, 2 mM glutamine, and 1000 U/mL penicillin–streptomycin (P/S) for 2 weeks. Primary cortical neurons from embryonic day 17 mice were cultured. In brief, cortices were dissected and incubated in ice-cold HBSS (LB003-02; Welgene, Gyeongsan, Korea) solution and dissociated in accumax (#A7089; Sigma Aldrich, St. Louis, MO, USA) for 15 min at 37 °C. The cultures were rinsed twice in HBSS. Mouse neurons were resuspended in a neurobasal medium (#21103049; Gibco, Waltham, MA, USA) containing 2% B27 (#17504; Gibco), 1% sodium pyruvate, and 1% P/S. Cells were filtered through a 70 um cell strainer (SPL, 93070), plated on culture plates, and maintained at 37 °C in a humidified 5% CO_2_ incubator. The medium was changed every 3 days; then, after 12–13 days in vitro, cells were used for experiments. Primary glial cells or cortical neurons were transfected with 100 nM miR-control, 100 nM has-miR-485-3p mimic, or 100 nM miR-485-3p ASO using TransIT-X2^®^ Transfection Reagent (Mirus Bio). 

### 4.7. Luciferase Assays

The human CD36 3′-UTR containing the target site for miR-485-3p was amplified from cDNA by PCR amplification and inserted into the pMir-target vector (Addgene, Watertown, MA, USA). To construct the vector expressing CD36 mutants, cDNA was amplified using a mutant seed sequence in the 514-520 region of CD36 mRNA 3′-UTR as the template: 5′-GAUAAAGGAAUUAUUGAUACUG-3′. HEK293T cells in 96-well plates were co-transfected with pMir-CD 36-3′-UTR WT or pMir-CD 36-3′-UTR MT and pRL-SV40 vector (Addgene) and miR-485-3p using Lipofectamine 2000 (#11668-027; Carlsbad, CA, USA). Cells were harvested 48 h later, and the Dual Luciferase Assay System (#E1910; Promega; Madison, WI, USA) was used to measure the luciferase reporter activities.

### 4.8. In Vitro Binding Assay

Streptavidin magnetic beads (#11205D; Invitrogen) were prepared for the in vitro binding assay as follows. Beads (50 mL) were washed five times with 500 mL of 1× B&W buffer (5 mM Tris-HCl, pH 7.4; 0.5 mM EDTA; 1 M NaCl). After removing the supernatant, the beads were incubated with 500 mL of 1× B&W buffer containing 100 μg of yeast tRNA (# AM7119; Invitrogen) for 2 h at 4 °C. The beads were washed twice with 500 mL of 1× B&W buffer and incubated with 200 mL of 1× B&W buffer containing 400 pmol of biotin-miR-485-3p for 10 min at 25 °C. The supernatant was removed, and the beads were washed twice with 500 mL of 1× B&W buffer and collected with a magnetic stand. The miRNA-coated beads were incubated with 500 mL of 1× B&W buffer containing 1 μg of in vitro transcribed target mRNA overnight at 4 °C. The following day, the beads were washed with 1 mL of 1× B&W buffer five times and then resuspended in 200 mL of RNase-free water. Bound RNA was extracted with the QiaZol Lysis reagent (#79306; Qiagen, Hilden, Germany) as per the manufacturer’s instructions. The extracted RNA was quantified using the StepOnePlus real-time PCR system (REF: 4376592; Applied Biosystems Foster City, CA, USA).

### 4.9. Immunoblotting

Brain tissue, primary microglia, and cortical neuron cells were homogenized in ice-cold RIPA buffer (iNtRON Biotechnology, Seongnam, Korea) containing a protease/phosphatase inhibitor cocktail (#5872; Cell Signaling Technology, Danvers, MA, USA) on ice for 30 min. The lysates thus obtained were centrifuged at 13,000 rpm for 15 min at 4 °C, and supernatants were collected. The samples were separated by SDS–PAGE, transferred to PVDF membranes, and incubated with the following primary antibodies: rabbit anti-APP (#2452, 1:1000; Cell Signaling Technology), mouse anti-sAPPα (#11088, 1:1000; IBL, Fujioka, Japan), mouse anti-sAPPβ (#10321, 1:1000; IBL), mouse anti-CTFs (#802803, 1:1000; Biolegend, San Diego, CA, USA), rabbit anti-β-amyloid (1–42) (#14974, 1:1000; Cell Signaling Technology), mouse anti-NeuN (#MAB377, 1:1000; Millipore, Burlington, MA, USA), rabbit anti-cleaved caspase-3 (#9664, 1:1000; Cell Signaling Technology), goat anti-Iba1 (#ab5076, 1:1000; Abcam, Cambridge, UK), rabbit anti-IL-1β (#9722, 1:1000; Abcam), mouse anti-TNF-α (#sc-52746; 1:1000; Santa Cruz Biotechnology, Dallas, TX, USA), rabbit anti-NF-κB (p65) (#8242, 1:1000; Cell Signaling Technology), mouse anti-TauC3 (#AHB0061, 1:1000; Thermo Fisher, Waltham, MA, USA), rabbit anti-CD36 (#ab133625, 1:1000; Abcam), and anti-β-actin (#sc-47778; Santa Cruz, Dallas, Texas 75220). The results were visualized using an enhanced chemiluminescence system and quantified by densitometric analysis (ImageJ software, NIH). 

### 4.10. Insoluble Aβ Extraction

Brain tissue samples were homogenized with RIPA buffer containing the protease/phosphatase inhibitor on ice, followed by centrifugation at 12,000 rpm for 15 min. The supernatants were collected. To obtain the insoluble fraction from brain tissue, the pellet of brain lysates was lysed in insoluble extraction buffer (50 mM Tris-HCl (pH 7.5) + 2% SDS) containing a protease/phosphatase inhibitor cocktail on ice for 30 min. The lysates were centrifuged at 4 °C for 15 min at 13,000 rpm. Protein was quantified using a bicinchoninic acid (BCA) assay kit (#5000116; Bio-Rad Laboratories, Hercules, CA, USA) and adjusted to the same final concentration. After denaturation, the lysates were processed for immunoblotting to measure insoluble Aβ content.

### 4.11. Immunohistochemistry

Brains of oligonucleotide-injected 8- or 10-month-old 5XFAD mice were removed, postfixed, and embedded in paraffin. Coronal sections (10 μm thick) through the infarct were cut using a microtome and mounted on slides. The paraffin was removed, and the sections were washed with PBS-T and blocked in 10% bovine serum albumin for 2 h. Thereafter, the following primary antibodies were applied: purified mouse anti-β-Amyloid (1–16; #803001, 1 μg/mL; Biolegend), rabbit anti-β-amyloid (1–42; #14974s, 1:100; Cell Signaling Technology), rabbit anti-Iba1 (#019-19741, 2 μg/mL; Wako, Osaka, Japan), goat anti-Iba1 (#ab5076, 2 μg/mL; Abcam), rabbit anti-CD68 (#ab125212, 1 μg/mL; Abcam), rat anti-CD36 (#ab80080, 1:100; Abcam), mouse anti-TNF-α (#sc-52746, 1:100; Santa Cruz, Dallas, Texas 75220), rabbit anti-IL-1β (#ab9722, 1 μg/mL; Abcam), rabbit anti-cleaved caspase-3 (#9662S, 1:300; Cell Signaling Technology), mouse anti-NeuN (#MAB377, 10 μg/mL; Millipore), rabbit anti-Synaptophysin (#ab14692, 1:50; Abcam), and rabbit anti-PSD-95 (#2507, 1:50; Cell Signaling Technology). Images were obtained using a confocal microscope (Leica DMi8).

For the lenti-miR-485-3p transduction, cells were fixed, and immunocytochemistry was performed to observe the levels of Aβ, p-tau, cleaved tau, synaptic marker, and ASC after 30 h of lenti-miR-485-3p virus transduction. Thereafter, the following primary antibodies were applied: rabbit anti-β-amyloid (1–42) (#805501, 1:50; Biolegend), mouse anti-cleaved tau (#AHB0061, 1:50; Invitrogen), mouse anti-phospho-tau (#MN1050, 1:50; Invitrogen), rabbit anti-PSD-95 (#2507, 1:200; Cell signaling), rabbit anti-Synaptophysin (#ab14692, 1:100; Abcam), and rabbit anti-ASC (#AB-25B-0006, 1:200; AdipoGen). Images were obtained using a confocal microscope (Leica DMi8). During confocal imaging, images were randomly taken; approximately 10 cells on average were distributed in one image. Quantification of the Aβ 1–42 (lenti-control *n* = 16, lenti-miR-485-3p *n* = 17), phospho-tau (lenti-control *n* = 13, lenti-miR-485-3p *n* = 15), cleaved tau (lenti-control *n* = 15, lenti-miR-485-3p *n* = 22), and synaptophysin and PSD-95 (synaptophysin, lenti-control *n* = 5, lenti-miR-485-3p *n* = 6; PSD-95, lenti-control *n* = 9, lenti-miR-485-3p *n* = 17) was used to analyze the intensity of the image corresponding to each value of *n* through Image J. 

### 4.12. ELISA Assay

Twenty-four hours after lenti-miR-485-3p transduction in mouse primary glial cells, ELISA (Mouse IL-1 beta/IL-1F2 Quantikine ELISA Kit (#MLB00C, R&D Systems) was performed using culture media to observe IL-1β release. 

### 4.13. Preparation of Aβ 1−42 Oligomer

The Aβ1–42 hexafluoroisopropanol (HFIP) peptide (#AS-64129) was obtained from AnaSpec (Fremont, CA, USA). The Aβ 1−42 oligomer (oligomeric Aβ, oAβ) synthetic human Aβ1–42 was dissolved in DMSO to a stock concentration of 5 mM subsequently diluted to 100 μM in serum-free DMEM and incubated at 37 °C for 24 h. oAβ content was confirmed by SDS-PAGE.

### 4.14. In Vitro Phagocytosis Assays

BV2 microglial cells (2 × 10^5^) were plated in six-well plates and incubated overnight. Cells were transfected using a TransIT-X2^®^ Transfection Reagent (Mirus Bio, Cat#MIR6000) according to the manufacturer‘s instructions and treated with oAβ for 4 h at a final concentration of 1 μM. In some cases, anti-CD36 was applied to the media with oAβ. After 4 h, the medium was collected from the BV2 microglial culture. Levels of human Aβ (1-42) in the supernatant were measured using the human Aβ42 ELISA kit (Invitrogen, Cat#KHB3441), according to the manufacturer’s instructions.

Microglial phagocytosis was verified by fluorescence microscopy. Coverslips were coated with poly-l-lysine before plating 8 × 10^4^ primary microglia per coverslip resting in the wells of a 24-well plate overnight. The cells were transfected using the TransIT-X2^®^ Transfection Reagent (Mirus Bio; Madison, WI, USA) according to the manufacturer’s instructions and incubated in unlabeled oAβ for 4 h at a final concentration of 1 μM. After 4 h, the cells were washed with cold PBS. For Aβ uptake measurement, primary microglia were fixed with 100% methanol for 1 h at −20 °C, washed with PBS-T, and incubated at 4 °C with mouse anti-β-Amyloid 1–16 and rabbit anti-Iba1 (#019-19741, 2 μg/mL; Wako).

### 4.15. FACS Analysis

All staining was performed in the dark, and blocking was performed with BD Fc Block. Primary microglia were stained using Alexa 488-conjugated anti-mouse CD36 (#102607, 5 μg/mL; Biolegend) or isotype control Ab (#400923, 5 μg/mL; Biolegend) for 30 min at 4 °C. After 30 min, cells were washed with FACS buffer (PBS+1%). Data were analyzed using the CellQuest (BD Bioscience) and FlowJo software (Tree Star) packages.

### 4.16. Real-Time PCR

Total RNA was isolated using a kit applicable to both small and large RNA molecules (Macherey-Nagel, Dueren, Germany). cDNA was synthesized using a miScript II RT kit (Qiagen). Analysis of the expression of miR-485-3p was performed as TaqMan miRNA analysis using TOPreal qPCR 2X PreMIX (Enzynomics, Daejeon, Korea) on a CFX connect system (Bio-Rad). The primers used were as follows: miR-485-3p forward: 5′-CATACACGGCTCTCCTCTCTAAA-3′, probe: FAM-CGAGGTCGACTTCCTAGA-NFQ. *RNU6* forward: 5′- GCTTCGGCAGCACATATACTAAAAT-3′, reverse: 5′- GAATCGAGCACCAGTTACG-3′, probe: FAM-CGAGGTCGACTTCCTAGA-NFQ. Relative gene expression was analyzed using the 2^−∆∆ct^ method.

### 4.17. Behavior Tests

The Y-maze consisted of three black, opaque, plastic arms (30 cm × 8 cm × 15 cm), set at 120° from each other. The 8-month-old 5XFAD mice were placed at the center of the Y-maze and allowed to explore all three arms. The number of arm entries and the number of trials (a shift is 10 cm from the center, entries into three separate arms) were recorded to calculate the percentage of alternation. An entry was defined as all four limbs are within a Y-maze arm, in which a spontaneous alternation occurs when a mouse enters a different arm of the maze in each of the three consecutive arm entries. Alternation behavior was defined as follows: ([number of trials]/[number of arm entries] − 2) × 100. The passive avoidance chamber was divided into a white (light) and a black (dark) compartment (41 cm × 21 cm × 30 cm). The light compartment contained a 60 W electric lamp. The floor of the dark compartment contained a number of 2 mm stainless steel rods spaced 5 mm apart. The test lasted 3 d. On the first day, mice were adapted to the bright zone for 5 min. The second day comprised the training phase, which consisted of two steps: In the first step, mice were placed in the light zone and then moved to the dark zone twice. One hour after the first step, mice were placed in the light compartment. The door separating the two compartments was opened 30 s later, and, after mice entered the dark compartment, the door was closed, and an electrical foot shock (0.3 mA/10 g) was delivered through the grid floor for 3 s. If a mouse did not enter the dark zone for more than 5 min, it was considered to have learned avoidance. The training was performed up to five times. Twenty-four hours after training, the mice were placed in the light chamber for testing. Latency was defined as the time it took for a mouse to enter the dark chamber after the door separating the two compartments opened. The time taken for a mouse to enter the dark zone and exit to the bright zone was defined as its TDC (time spent in the dark compartment).

#### Data Analysis

All data are presented as mean ± SD or S.E.M. The statistical significance of the values obtained across the two groups was determined using an unpaired *t*-test. Statistical tests were performed using GraphPad Prism 8 and R (version 4.0.0). The behavior test results were assessed using nonparametric statistical procedures.

## Data Availability

The data generated during this study is available upon request.

## Supplementary Materials

The following are available online at https://www.mdpi.com/1422-0067/22/23/13136/s1.
