# Correction: Koh et al. Targeting MicroRNA-485-3p Blocks Alzheimer’s Disease Progression. *Int. J. Mol. Sci.* 2021, *22*, 13136

**DOI:** 10.3390/ijms23073566

**Published:** 2022-03-25

**Authors:** Han Seok Koh, SangJoon Lee, Hyo Jin Lee, Jae-Woong Min, Takeshi Iwatsubo, Charlotte E. Teunissen, Hyun-Jeong Cho, Jin-Hyeob Ryu

**Affiliations:** 1 BIORCHESTRA Co., Ltd., 17, Techno 4-ro, Yuseong-gu, Daejeon 34013, Korea; kohhan@biorchestra.com (H.S.K.); hohosaraly@biorchestra.com (H.J.L.); mjw@biorchestra.com (J.-W.M.); 2 Department of Infection Biology, Faculty of Medicine, University of Tsukuba, Ibaraki 305-8577, Japan; frank.sj.lee@gmail.com; 3 Department of Neuropathology, Graduate School of Medicine, The University of Tokyo, Tokyo 113-0033, Japan; iwatsubo@m.u-tokyo.ac.jp; 4 Neurochemistry Laboratory and Biobank, Department of Clinical Chemistry, Amsterdam Neuroscience, Amsterdam University Medical Centers, 1081 HV Amsterdam, The Netherlands; c.teunissen@amsterdamumc.nl; 5 Department of Biomedical Laboratory Science, College of Medical Science, Konyang University, Daejeon 35365, Korea; 6 BIORCHESTRA US Inc., 245 Main St., Cambridge, MA 02142, USA

The authors wish to make the following corrections to this paper [1]. In the Affiliations:

Dr. SangJoon Lee has been requested by Ulsan National Institutes of Science and Technology (UNIST) that his affiliation of UNIST be removed from the Affiliations, because Dr. Lee’s position was not officially assigned by UNIST when the paper was published.

The corrected affiliation section should be: SangJoon Lee^2^: Department of Infection Biology, Faculty of Medicine, University of Tsukuba, Ibaraki 305-8577, Japan. 

The authors apologize for any inconvenience caused and state that the scientific conclusions are unaffected. The original publication has also been updated.

## References

[1] Koh H.S., Lee S., Lee H.J., Min J.-W., Iwatsubo T., Teunissen C.E., Cho H.-J., Ryu J.-H. (2021). Targeting MicroRNA-485-3p Blocks Alzheimer’s Disease Progression. Int. J. Mol. Sci..

