# Supplementary material for: Targeting MicroRNA-485-3p Blocks Alzheimer’s Disease Progression
Source: Int J Mol Sci. 2021 Dec 4;22(23):13136. doi: 10.3390/ijms222313136 (PMC8658496; doi:10.3390/ijms222313136)
Supplement: Supplementary file 1 [file ijms-22-13136-s001.zip › Supplementary Figures.pdf]

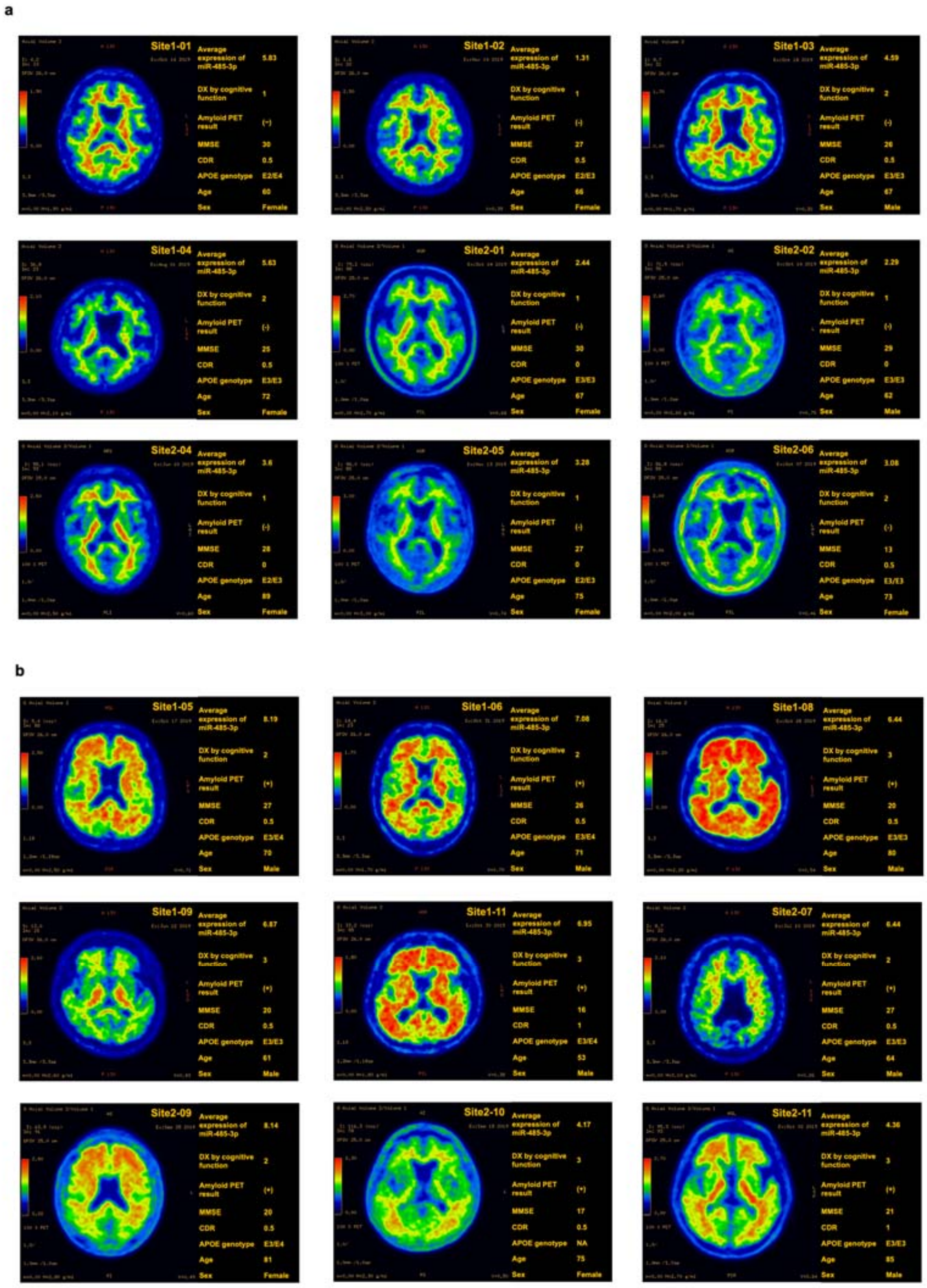

Supplementary Figure S1. Amyloid PET images and profiling of subjects with amyloid PET-negative and -positive results. (A and B) Representative images of amyloid PET-negative samples (A) and -positive samples (B). Amyloid PET images were provided by Gyeongsang National University Hospital (Site 1) and Eulji Medical Center (Site 2). The images for site 1 were obtained using GE Discovery PET/CT 710 as a scanner and florbetamol as a tracer. The images for site 2 were obtained using GE Discovery PET/CT 690 as a scanner and florbetaben as a tracer. Average expression of miR-485-3p, mini-mental state examination (MMSE), clinical dementia rate (CDR), apolipoprotein E (APOE) genotype, age, and sex are described in each sample.

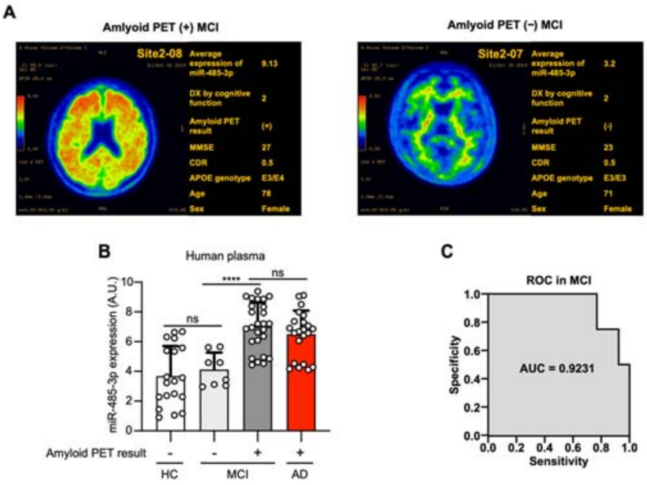

Supplementary Figure S2. miR-485-3p expression was upregulated in positive Aβ PET MCI patients compared with that in negative Aβ PET MCI patients. (A) Representative amyloid PET images and profiling of patients with the highest (left side) and lowest (right side) miR-485-3p expression levels among MCI patients. Average expression of miR-485-3p, mini-mental state examination (MMSE), clinical dementia rate (CDR), apolipoprotein E (APOE) genotype, age, and sex are described for each sample. (B) Expression level of miR-485-3p obtained from healthy control (n = 19), amyloid PET-negative MCI patients (n = 8), amyloid PET-positive MCI patients (n = 26), and amyloid PET-positive AD patients (n = 22). (C) ROC (receiver operating characteristic) analysis result of miR-485-3p expression with or without amyloid PET among MCI patients. ns, not significant; ****P < 0.0001 (two-tailed t-test).

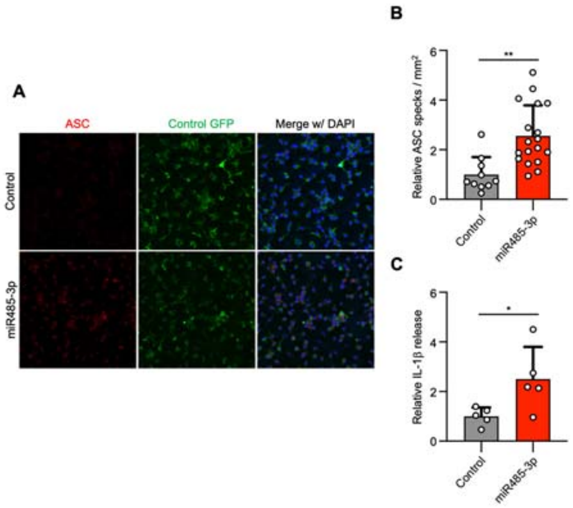

Supplementary Figure S3. miRNA-485-3p triggers inflammasome-mediated inflammatory responses. (A and B) Primary mouse microglia were transduced with lentivirus-derived control or miR485-3p. The cells were examined by immunocytochemistry using the ASC antibody (A). Data are representative of three independent experiments. Quantification of ASC specks from (B). (Data, obtained across three independent experiments, are expressed as mean ± SD) (C) Release of IL-1β in primary mouse microglia after lenti-control or lenti-miR485-3p transduction. (Data, obtained across three independent

experiments, are expressed as mean ± SD). *P < 0.05; **P < 0.01 (two-tailed *t*-test).

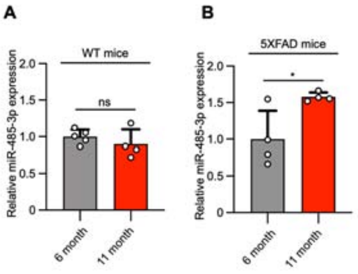

Supplementary Figure S4. Expression of miR485-3p is increased in 5XFAD mice. (A and B) The small RNAseq on the frontal cortex of the 5XFAD mice was performed/ For NGS, Illumina's Hiseq2000 instrument was used. miRNA profiling was performed using the miRdeep2 tool and the results were used to obtain raw reads from Fastq files. Afterwards, the raw read was normalized by the TMM method. Relative gene expressions of miR-485-3p in 6-month-old and 11-month-old WT mice (A) and 5XFAD mice (B). Data, obtained across three independent experiments, are expressed as mean ± SD from three independent experiments. ns, not significant; *P < 0.05 (two-tailed *t*-test).

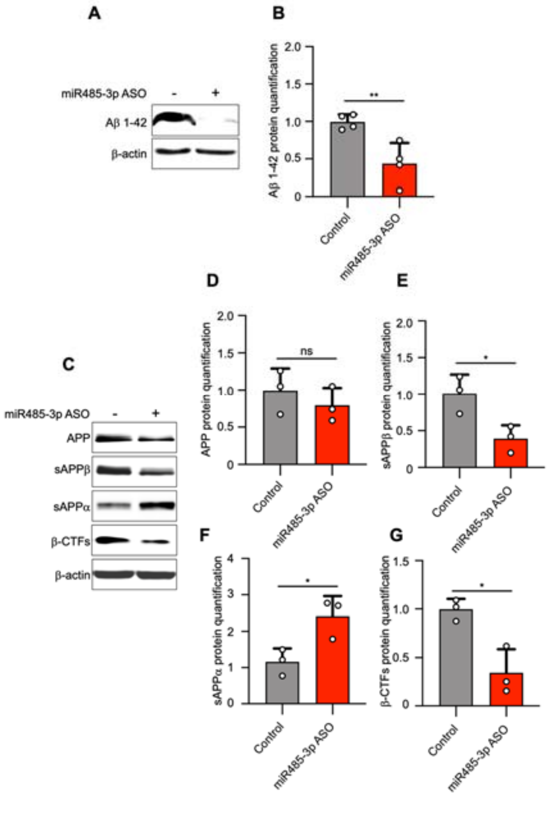

Supplementary Figure S5. miR485-3p ASO leads non-amyloidogenic pathway in 5XFAD mice. (A) Immunoblotting for insoluble Aβ fractions in the cortex region of control- or miR485-3p ASO-injected 8-month-old 5XFAD mice. Data are representative of four independent experiments. (B) Relative protein quantification of Aβ (1–42) obtained from (A). Data, obtained across four independent experiments, are expressed as mean ± SD. (C), Immunoblotting for APP, sAPPβ, sAPPa, and β-CTFs in the cortex region of control- or miR485-3p ASO-injected 8-month-old 5XFAD mice. Data are representative of three independent experiments. (D–G) Relative protein quantification of APP (D), sAPPβ (E), sAPPα (F), and β-CTFs (G) obtained

from (C). Data, obtained across three independent experiments, are expressed as mean ± SD. ns, not significant; *P < 0.05; **P < 0.01 (two-tailed *t*-test).
